# Annotations

**Published:** 1887-09-10

**Authors:** 


### Annotations.

The *Spectator* undoubtedly takes sides, and apparently would almost rather patients died than were cured by Pasteur's method. We sympathise with the feeling which hates to injure an animal even for the sake of benefiting a human being. If, however, the facts be in favour of Pasteur, we should consider it right to sacrifice the animal to the man's well-being, just as we sacrifice it now to his comfort and health when we kill it for food. But that is the very point at issue: are the facts entirely or mainly on the side of the famous Frenchman? The Pasteur commission, consisting of some of the most eminent and trusted English men of science, says they are. Nevertheless, the Austrian Government has withdrawn the subvention granted last year to the Vienna General Hospital to defray the cost of inoculation on the Pasteur system. The failures have been so numerous as to discourage competent judges from further supporting the method. The news of the death of Lord Doneraile comes to impart a melancholy confirmation of the views of the Austrian Government. Who is to be believed—Mr. Victor Horsley and his brother commissioners, or the hospital officials in Vienna, who must be ultimately responsible for the decision of their Government? It is simply a question of facts. What do these say? They do not up to the present time say anything authoritative: or if they do, the authority is on the side of failure. Where so many treated cases die, the method cannot be one of absolute cure. There cannot be one law for the scientist and another for the barbarian. Both alike must submit to the only authoritative test—the test of fact. Just as when the faith-healers affirm that a miracle has been performed, we say, "Let us see sufficient proof of the miracle and we will believe it"; so when the man of science says he has got a cure for a certain disease, we argue, "Produce your cured cases: let them be so evident, numerous, and unfailing that the uninitiated as well as the initiated may see and be convinced. If the cure be reliable there should be no need for commissions, inquiries, and reasonings: the hard facts would appeal to all. When a man has got typhoid fever the disease is palpable: when he has recovered his recovery is equally undoubted. His child of six understands that as well as his physician of sixty. Let us have on the Pasteur controversy an array of facts as evident and as undeniable as a series of cases of typhoid fever. Then there will be materials for a judgment—not before.

More Pasteur Failures.

At the British Pharmaceutical Conference just held in Manchester, Professor Leech, who read a paper on Pharmacy, pointed to a time when it may be anticipated that medical men will cease entirely to be compounders of drugs, and chemists will no longer be mere distributors, but guarantors of purity. For the sake of the patient, the doctor, and the druggist alike, it would be well if things were already as the Professor hopes they sometime will be. Nobody can say now that the poorer classes, or even the less wealthy amongst the middle classes, are supplied with drugs of ideal perfection. But, if perfection be aimed at anywhere it should surely be in the management of the sick, and in the medicines in which they hope to find relief and restoration to health. It is impossible to imagine anything more grievous to the conscientious and sympathetic doctor than the knowledge that the prescription, which he has constructed with so much anxious thought and care, may contain in high perfection few or none of the elements which he wishes it to contain; and may on the other hand be so mixed with impurities that not only can there be none of the positive physiological effects he desires, but there may be certain decided physiological effects which he does not desire, and which may be injurious to his patient. Of course it is a matter of supply and demand, of the power of people to pay for the best. One solution of the difficulty would be the diminution of the costl of compounded physic. Against that the druggist avers that he must live. In order to this it might be well to raise the standard of examination, and so to diminish the number of chemists. In addition let all dispensing be done by chemists. There would thus be an amount of business for each which might warrant a large diminution of prices. The doctor could then keep to his own department, and the chemist to his, without overlapping. At present there is a system of mutual poaching going on. The doctor dispenses, thereby dragging his partridge-net over the chemist's new-mown fields. The chemist prescribes, thus ruining the doctor's choicest preserves; and they metaphorically shoot one another all the year round—a feud as unnecessary as it is miserable.

Doctors and Druggists.

The people of Leicester have the courage of their convictions. Official returns made last week show that during the past twelve months, out of 5,000 births, only 550 children were vaccinated. Of these no more than thirty-nine were vaccinated by private medical men. It is clear therefore that the wealthier and better-educated classes are fully as determined to have nothing to do with vaccination as their poorer neighbours. For our part we do not altogether regret the attitude taken up by the Leicester people. The faith of our forefathers, medical as well as theological, requires to be brought to the test of present-day reason and present-day experience occasionally. The anti-vaccinators have long been arguing eloquently on speculative grounds, and the pro-vaccinators are under the impression that they have so overwhelmed their opponents with hard facts that if they are not pounded to a metaphorical jelly they ought to be. Leicester has valiantly come to the rescue, and put itself bodily into the crucible for an experiment on a large scale. Within the next ten years there may be an epidemic which will reach Leicester—we hope there will not, but there may be—and then there will either be much triumphant jubilation among its people, or "mourning, lamentation, and woe." We cannot prevent the experiment being made. We look forward to it with a fearful kind of interest which is anything but merely scientific.

Anti-vaccinators.

